# Supplementary material for: Renal Outcome of IgM Nephropathy: A Comparative Prospective Cohort Study
Source: J Clin Med. 2021 Sep 16;10(18):4191. doi: 10.3390/jcm10184191 (PMC8466757; doi:10.3390/jcm10184191)
Supplement: Supplementary file 1 [file jcm-10-04191-s001.zip › jcm-1367151-supplementary.pdf]

Supplementary Materials

Supplementary Table S1. Associations between each variable and renal outcome from longitudinal data (baseline and follow-up).

	**Serum creatinine**		**eGFR**		**UPCR**	
	**β (se)**	***p* value**	β (se)	*p* value	β (se)	*p* value
Clinical findings						
Age	0.011 (0.006)	0.083	-1.20 (0.125)	<0.001	0.065 (0.017)	<0.001
Sex, female	-0.311 (0.192)	0.107	0.783 (5.085)	0.878	-0.062 (0.574)	0.914
Alcohol	0.171 (0.208)	0.412	-4.63 (6.305)	0.465	-0.260 (0.640)	0.685
Smoking						
Non smoker	1		1		1	
Past smoker	-0.189 (0.284)	0.508	-14.3 (8.720)	0.114	2.931 (1.271)	0.026
Current smoker	-0.114 (0.210)	0.586	-0.276 (7.809)	0.972	-0.373 (0.704)	0.597
Hypertension	0.304 (0.242)	0.212	-19.3 (4.964)	<0.001	0.457 (0.596)	0.443
Diabetes	0.287 (0.284)	0.316	-9.89 (7.823)	0.215	0.366 (0.942)	0.699
SBP	0.021 (0.006)	<0.001	-0.649 (0.156)	<0.001	0.010 (0.018)	0.577
DBP	0.024 (0.010)	0.025	-0.298 (0.267)	0.271	-0.031 (0.033)	0.351
WBC	-0.018 (0.038)	0.638	0.286 (1.109)	0.797	0.325 (0.126)	0.01
Hb	-0.135 (0.045)	0.003	8.198 (1.132)	<0.001	0.236 (0.129)	0.069
HbA1c	0.368 (0.171)	0.033	-15.6 (4.871)	0.003	0.866 (0.561)	0.128
BUN	0.068 (0.005)	<0.001	-2.96 (0.202)	<0.001	0.090 (0.029)	0.003
ESR	0.007 (0.003)	0.018	-0.357 (0.103)	0.001	0.073(0.013)	<0.001
Hs-CRP	0.619 (0.298)	0.041	-3.30 (1.556)	0.079	-0.102 (0.102)	0.381
Total protein	0.066 (0.062)	0.288	-0.145 (1.955)	0.941	-2.25 (0.181)	<0.001
Albumin	0.027 (0.078)	0.731	3.945 (2.283)	0.085	-3.10 (0.214)	<0.001
Total cholesterol	-0.002 (0.001)	0.005	0.063 (0.020)	0.002	0.022(0.003)	<.0001
Ferritin	0.003 (0.001)	<0.001	-0.039 (0.014)	0.008	0.005 (0.001)	0.001
C3	-0.008 (0.004)	0.035	0.240 (0.093)	0.014	0.010 (0.013)	0.483
C4	0.009 (0.008)	0.280	-0.082 (0.201)	0.686	0.055 (0.021)	0.01
IgG	4.5E-04 (2.3E-04)	0.045	-0.019 (0.006)	0.001	-0.005(0.001)	<0.001
IgA	1.3E-03 (9.4E-04)	0.182	-0.046 (0.024)	0.059	0.003 (0.003)	0.305
IgM	-0.004 (0.002)	0.066	9.2E-04 (6.2E-03)	0.883	0.002 (0.002)	0.173
Ig E	-3.8E-06 (3.0E-05)	0.901	-0.004 (0.004)	0.442	0.003 (0.001)	0.002
Treatment						
RAAS blocker	-0.428 (0.260)	0.103	-1.86 (5.724)	0.746	-1.33 (0.687)	0.054
Furosemide	0.164 (0.191)	0.393	-8.35 (5.550)	0.134	5.678 (0.657)	<0.001
Glucocorticoids	0.023 (0.199)	0.906	-9.65 (5.256)	0.068	4.232 (0.588)	<0.001
Rituximab	0.243 (1.331)	0.856	-39.8 (33.87)	0.449	1.568 (4.635)	0.735
Other immunosuppressants	-0.450 (0.335)	0.182	5.548 (12.06)	0.653	1.048 (1.177)	0.375
**Pathologic findings**						
Glomerulosclerosis	0.012 (0.004)	0.004	-0.770 (0.080)	<0.001	-0.034(0.015)	0.026
Mesangial matrix expansion		0.417		0.116		0.371
0, negative	1		1		1	
1, trace	-0.304 (0.266)	0.256	3.518 (6.181)	0.570	-0.278 (0.739)	0.707
2, mild	-0.118 (0.296)	0.690	0.862 (7.544)	0.909	-2.25 (0.810)	0.006

3, moderate	-0.515 (0.440)	0.246	3.647 (22.17)	0.877	1.020 (2.153)	0.636
4, marked	1.059 (0.769)	0.259	-51.8 (17.07)	0.049	2.356 (2.751)	0.392
Mesangial cell proliferation		0.470		0.69		0.021
0, negative	1		1		1	
1, trace	-0.463 (0.263)	0.081	5.584 (6.160)	0.366	0.386 (0.774)	0.619
2, mild	0.034 (0.301)	0.910	-2.57 (7.573)	0.735	-1.92 (0.702)	0.007
3, moderate	-0.206 (0.438)	0.641	-19.3 (18.50)	0.346	1.730 (3.558)	0.651
Crescent	0.011 (0.011)	0.331	-0.75 (0.25)	0.003	-0.072 (0.038)	0.127
Interstitial fibrosis		0.019		<0.001		0.561
0, negative	1		1		1	
1, trace	0.094 (0.163)	0.564	-21.8 (6.040)	<0.001	0.821 (0.877)	0.35
2, mild	0.547 (0.189)	0.004	-42.3 (5.785)	<0.001	-1.50 (0.775)	0.054
3, moderate	1.977 (0.789)	0.019	-66.3 (7.853)	<0.001	-0.743 (0.914)	0.417
4, marked	3.172 (2.484)	0.203	-95.4 (36.82)	0.011	0.657 (6.650)	0.921
Tubular atrophy		0.025		<0.001		0.98
0, negative	1		1		1	
1, trace	0.018 (0.161)	0.910	-18.7 (5.929)	0.002	0.443 (0.813)	0.586
2, mild	0.520 (0.184)	0.005	-39.7 (5.929)	<0.001	-1.32 (0.813)	0.104
3, moderate	1.879 (0.870)	0.042	-64.6 (8.105)	<0.001	-0.993 (1.111)	0.372
4, marked	3.530 (1.123)	0.086	-94.2 (20.28)	<0.001	0.198 (2.780)	0.943
Acute tubular necrosis		0.943		0.177		0.752
0, negative	1		1		1	
1, trace	0.648 (0.460)	0.228	-39.3 (17.96)	0.091	3.273 (3.388)	0.388
2, mild	2.937 (1.678)	0.111	-48.1 (9.347)	<0.001	0.897 (1.334)	0.507
3, moderate	1.788 (0.567)	0.011	-61.8 (8.107)	<0.001	6.397 (2.744)	0.042
Arterial intimal hyalinosis		0.912		0.044		0.944
0, negative	1		1		1	
1, trace	0.059 (0.428)	0.890	-20.7 (10.51)	0.063	1.118 (1.278)	0.382
2, mild	-0.201 (0.576)	0.727	-12.6 (15.90)	0.449	0.734 (1.720)	0.670
3, moderate	0.335 (0.806)	0.678	-36.2 (22.12)	0.197	-2.260 (2.408)	0.349
Fibrous wall thickening		0.439		0.632		0.639
0, negative	1		1		1	
1, trace	1.389 (1.059)	0.206	-17.6 (11.64)	0.145	-1.420 (1.163)	0.224
2, mild	-0.036 (0.238)	0.878	-11.7 (8.447)	0.173	0.206 (0.969)	0.832
3, moderate	0.143 (0.228)	0.531	-20.6 (8.808)	0.026	0.618 (1.090)	0.571
4, marked	0.678 (0.759)	0.415	-50.5 (17.19)	0.050	-2.000 (2.783)	0.473

* Mixed effects models with time (baseline, 1st visit, 2nd visit), factor, time*factor as fixed effects were performed with random intercept and slopes for each individual value, allowing unstructured correlation between the random effects. BUN, blood urea nitrogen; DBP, diastolic blood pressure; ESR, erythrocyte sedimentation rate; FSGS, focal segmental glomerulosclerosis; Hb, hemoglobin; HbA1c, hemoglobin A1c; hs-CRP, high sensitivity c-reactive protein; IgMN, IgM nephropathy; MCD, minimal change disease; MsPGN, mesangial proliferative glomerulonephritis; RAAS, renin-angiotensin-aldosterone system; SBP, systolic blood pressure; UPCR, urine protein-to-creatinine ratio; WBC, white blood cell.

Supplementary Table S2. Associations between each variable and renal outcome from longitudinal data (baseline and follow-up) in IgMN patients.

	Serum creatinine				eGFR				UPCR			
	model 1		model 2		model 1		model 2		model 1		model 2	
	B (se)	*p* value	β (se)	*p* value	β (se)	*p* value	β (se)	*p* value	β (se)	*p* value	β (se)	*p* value
Clinical findings												
Age	0.008 (0.009)	0.435			-1.15 (0.266)	<0.001	-0.463 (0.237)	0.055	0.103 (0.043)	0.019	0.041 (0.034)	0.234
Female	-0.400 (0.265)	0.133			8.830 (8.291)	0.289			-0.100 (1.149)	0.930		
Alcohol	0.811 (0.414)	0.059			-16.7 (10.67)	0.130			-0.712 (1.294)	0.584	-0.712 (1.294)	0.584
Smoking		0.722				0.041		0.143		0.092		
Non smoker	1				1				1			
Past smoker	0.010 (0.530)	0.984			-32.7 (10.77)	0.006	-42.6 (22.29)	0.062	7.117 (3.500)	0.045		
Current smoker	0.035 (0.363)	0.924			-10.7 (13.18)	0.427	3.436 (7.706)	0.657	-0.047 (2.047)	0.982		
Hypertension	0.241 (0.279)	0.390			-17.6 (7.964)	0.030	-3.35 (7.687)	0.665	0.154 (1.246)	0.902		
Diabetes	1.747 (0.745)	0.050			-29.3 (16.59)	0.122			1.557 (2.510)	0.551		
SBP	0.026 (0.008)	0.003	0.010 (0.006)	0.082	-0.639 (0.236)	0.013	-0.051 (0.179)	0.778	0.018 (0.039)	0.641		
DBP	0.024 (0.014)	0.100			-0.224 (0.483)	0.651			-0.038 (0.065)	0.568		
WBC	0.019 (0.056)	0.739			-1.66 (1.703)	0.336			0.475 (0.272)	0.085		
Hb	-0.160 (0.074)	0.042	-0.003 (0.042)	0.947	7.838 (1.856)	<0.001	1.625 (1.581)	0.308	0.292 (0.335)	0.386		
HbA1c	0.367 (0.393)	0.356			-17.8 (9.447)	0.139			0.548 (1.343)	0.686		
BUN	0.095 (0.006)	<.0001	0.041 (0.008)	<0.001	-3.06 (0.289)	<0.001	-1.25 (0.313)	<0.001	0.061 (0.050)	0.232		
ESR	0.024 (0.008)	0.003	-0.005 (0.004)	0.159	-0.384 (0.177)	0.036	0.274 (0.160)	0.094	0.098 (0.027)	0.001	-0.043 (0.027)	0.119
hs-CRP	0.399 (0.237)	0.114			-7.65 (6.117)	0.276			-0.427 (0.714)	0.551		
Total protein	-0.120 (0.106)	0.259			4.814 (3.516)	0.178			-2.38 (0.391)	<0.001	0.292 (1.074)	0.786
Albumin	-0.210 (0.148)	0.161			8.486 (4.073)	0.041	-3.85 (4.715)	0.418	-4.06 (0.518)	<.0001	-3.18 (1.304)	0.016
Total cholesterol	-0.002 (0.002)	0.214			0.076 (0.037)	0.058			0.029 (0.007)	<0.001	5.6E-03(9.0E-03)	0.534
Ferritin	0.003 (0.001)	0.004	2.1E-05 (4.5E-04)	0.963	-0.054 (0.026)	0.056			0.004 (0.003)	0.199		
C3	-0.004 (0.006)	0.562			0.352 (0.171)	0.042	0.114 (0.142)	0.426	0.017 (0.029)	0.569		

C4	0.019 (0.013)	0.157			-0.322 (0.459)	0.504			0.072 (0.058)	0.215		
IgG	1.6E-04 (3.6E-04)	0.663			-0.056 (0.043)	0.193			-0.005 (0.002)	0.006	4.6E-04 (1.9E-03)	0.811
IgA	0.003 (0.001)	0.062			-0.007 (0.010)	0.482			0.005 (0.007)	0.482		
IgM	4.6E-05 (5.5E-04)	0.935			0.002 (0.006)	0.750			0.002 (0.002)	0.265		
IgE	5.2E-04 (3.3E-04)	0.132			-0.006 (0.005)	0.247			0.005 (0.002)	0.014	2.2E-04 (5.6E-04)	0.693
IgMN subtype		0.153				0.007		0.424		0.016		0.387
MCD-like	1				1				1			
FSGS-like	0.290 (0.394)	0.464			-21.4 (11.43)	0.067	-1.49 (10.48)	0.887	-2.14 (1.742)	0.225	-0.568 (1.789)	0.752
MsPGN-like	-0.420 (0.334)	0.215			19.87 (10.21)	0.058	10.09 (9.932)	0.314	-8.11 (1.366)	<.0001	-2.27 (1.993)	0.258
Treatment												
RAAS blocker	-0.277 (0.305)	0.367			-5.90 (9.328)	0.529			0.000 (1.319)	>0.999		
Furosemide	0.882 (0.317)	0.007	0.185 (0.217)	0.395	-23.7 (9.165)	0.012	-2.06 (8.861)	0.817	5.116 (1.367)	0.001	-0.779 (1.483)	0.6
Glucocorticoids	0.090 (0.267)	0.735			-25.3 (8.111)	0.002	-10.4 (6.992)	0.143	4.031 (1.179)	0.001	-1.40 (1.288)	0.279
Rituximab	0.107 (1.763)	0.952			-33.8 (53.33)	0.527			1.659 (5.575)	0.767		
Other Immuno-suppressants	-0.427 (0.484)	0.381			-4.13 (19.88)	0.843			-0.052 (0.675)	0.939		

* Model 1: including factor, time (baseline and follow-up) and time*factor as fixed effects with random intercept and slopes for each individual value, allowing unstructured correlation between the random effects; Model 2: including variables with p <0.05 in model 1, time and follow-up period (month) as fixed effects with random intercept for each individual value, allowing unstructured correlation between the random effects.. BUN, blood urea nitrogen; DBP, diastolic blood pressure; ESR, erythrocyte sedimentation rate; FSGS, focal segmental glomerulosclerosis; Hb, hemoglobin; HbA1c, hemoglobin A1c; hs-CRP, high sensitivity c-reactive protein; IgMN, IgM nephropathy; MCD, minimal change disease; MsPGN, mesangial proliferative glomerulonephritis; RAAS, renin-angiotensin-aldosterone system; SBP, systolic blood pressure; UPCR, urine protein-to-creatinine ratio; WBC, white blood cell.

Supplementary Table S3. Univariate logistic regression for ≥20% decline in eGFR over time in IgMN patients.

	Crude OR (95% CI)	*p* value
IgMN subtype		
MCD like	1	
FSGS like	5.44 (0.77–38.26)	0.089
MsPGN like	1.26 (0.17–9.36)	0.824
Clinical findings		
Age	1.02 (0.98–1.06)	0.429
Sex		
Male	1	
Female	0.55 (0.14–2.19)	0.398
Alcohol		
No	1	
Yes	1.79 (0.37–8.74)	0.472
Smoking		
Non-smoker	1	
Past smoker	4.03 (0.42–38.94)	0.229
Current smoker	1.13 (0.17–7.65)	0.902
Hypertension		
No	1	
Yes	4.41 (1.09–17.77)	0.037
DM		
No	1	
Yes	0.55 (0.02–12.66)	0.709
SBP	1.02 (0.99–1.05)	0.235
DBP	0.99 (0.93–1.05)	0.686
WBC	0.93 (0.70–1.23)	0.605
Hb,	1.01 (0.74–1.38)	0.963
HbA1c	1.42 (0.41–4.89)	0.577
BUN	1.02 (0.99–1.05)	0.235
Creatinine	1.00 (0.65–1.54)	0.99
UPCR	0.98 (0.87–1.10)	0.723
ESR	0.98 (0.95–1.02)	0.32
hs-CRP	0.91 (0.42–1.96)	0.808
Total protein	1.19 (0.70–2.05)	0.52
Albumin	1.20 (0.63–2.30)	0.581
Total cholesterol	1.00 (0.99–1.00)	0.492
Ferritin	1.00 (0.99–1.00)	0.313
C3	1.00 (0.97–1.03)	0.893
C4,	1.02 (0.97–1.06)	0.532
Ig G (serum)	1.00 (1.00–1.00)	0.287
Ig A (serum)	1.00 (1.00–1.01)	0.552
Ig M (serum)	1.00 (1.00–1.00)	0.853
Ig E (serum)	1.00 (1.00–1.00)	0.64
Pathologic findings		
Glomerulosclerosis	1.01 (0.99–1.03)	0.428
Mesangial matrix expansion		
0, negative	1	
1, trace	0.83 (0.18–3.69)	0.802
2, mild	1.16 (0.21–6.30)	0.862
3, moderate	1.10 (0.03–38.64)	0.96

4, marked	1.01 (0.99–1.03)	0.428
Mesangial cell proliferation		
0, negative	1	
1, trace	0.80 (0.18–3.57)	0.771
2, mild	1.04 (0.19–5.56)	0.967
Crescent	1.00 (0.96–1.05)	0.866
Interstitial fibrosis		
0, negative	1	
1, trace	0.42 (0.02–11.48)	0.606
2, mild	5.44 (0.82–35.91)	0.079
3, moderate	5.18 (0.57–47.33)	0.145
4, marked	6.60 (0.05–815.4)	0.443
Tubular atrophy		
0, negative	1	
1, trace	0.52 (0.02–14.30)	0.699
2, mild	6.75 (1.03–44.46)	0.047
3, moderate	7.44 (0.79–70.13)	0.079
4, marked	3.19 (0.07–143.0)	0.55
Acute tubular necrosis		
0, negative	1	
2, mild	0.72 (0.03–18.51)	0.844
3, moderate	0.72 (0.03–18.51)	0.844
Arterial intimal hyalinosis		
0, negative	1	
1, trace	2.10 (0.28–15.97)	0.472
2, mild	3.26 (0.04–295.3)	0.608
Fibrous wall thickening		
0, negative	1	
1, trace	2.41 (0.30–19.56)	0.41
2, mild	1.08 (0.15–7.58)	0.937
3, moderate	4.02 (0.68–23.86)	0.126
4, marked	3.46 (0.03–350.9)	0.598
Treatment		
RAAS blocker		
No use	1	
Use	1.77 (0.39–8.06)	0.462
Furosemide		
No use	1	
Use	0.74 (0.16–3.38)	0.694
Glucocorticoids		
No use	1	
Use	2.08 (0.55–7.90)	0.284
Rituximab		
No use	1	
Use	2.91 (0.03–284.9)	0.648
Other immunosuppressants		
No use	1	
Use	7.76 (1.13–53.51)	0.038

* BUN, blood urea nitrogen; DBP, diastolic blood pressure; ESR, erythrocyte sedimentation rate; FSGS, focal segmental glomerulosclerosis; Hb, hemoglobin; HbA1c, hemoglobin A1c; hs-CRP, high sensitivity c-reactive protein; IgMN, IgM nephropathy; MCD, minimal change disease; MsPGN, mesangial proliferative glomerulonephritis; RAAS, renin-angiotensin-aldosterone system; SBP, systolic blood pressure; UPCR, urine protein-to-creatinine ratio; WBC, white blood cell.
